# Interactions Between Laminin Receptor and the Cytoskeleton During Translation and Cell Motility

**DOI:** 10.1371/journal.pone.0015895

**Published:** 2011-01-07

**Authors:** Lisa Venticinque, Kelly V. Jamieson, Daniel Meruelo

**Affiliations:** Gene Therapy Center, Cancer Institute and Department of Pathology, New York University School of Medicine, New York, New York, United States of America; Kings College London, United Kingdom

## Abstract

Human laminin receptor acts as both a component of the 40S ribosomal subunit to mediate cellular translation and as a cell surface receptor that interacts with components of the extracellular matrix. Due to its role as the cell surface receptor for several viruses and its overexpression in several types of cancer, laminin receptor is a pathologically significant protein. Previous studies have determined that ribosomes are associated with components of the cytoskeleton, however the specific ribosomal component(s) responsible has not been determined. Our studies show that laminin receptor binds directly to tubulin. Through the use of siRNA and cytoskeletal inhibitors we demonstrate that laminin receptor acts as a tethering protein, holding the ribosome to tubulin, which is integral to cellular translation. Our studies also show that laminin receptor is capable of binding directly to actin. Through the use of siRNA and cytoskeletal inhibitors we have shown that this laminin receptor-actin interaction is critical for cell migration. These data indicate that interactions between laminin receptor and the cytoskeleton are vital in mediating two processes that are intimately linked to cancer, cellular translation and migration.

## Introduction

The 37/67 kDa laminin receptor (LamR), originally discovered as a 67 kDa cell surface receptor for laminin-1 in the extracellular matrix (ECM) [1], [2], [3], has a dual function as a component of the translational machinery and a cell surface receptor. The relationship between the 37 kDa and 67 kDa forms of LamR is not completely understood. The 67 kDa form of LamR is predicted to be a dimer, but whether LamR forms a homo-dimer [4] or hetero-dimer [5], [6] has yet to be resolved. Amino acid composition analysis indicates that LamR exists as a homo-dimer at the cell surface [4]. Immunoblotting of detergent extracts indicates that LamR forms a hetero-dimer with galectin3 [5]. Additionally, post translational modifications have been suggested to stabilize the 67 kDa form and may be required for LamR association with the cell membrane [4], [5]. At the cell surface, LamR also acts as the receptor for several viruses including Sindbis virus [7], Venezuelan equine encephalitis virus [8] and Dengue virus [9], [10] as well as for prion proteins [11]. LamR, which is upregulated on a number of human cancers [12], [13], [14], [15], [16], plays a role in migration, tumor invasion and metastasis [17], [18] through interactions with laminin-1. Intracellularly, LamR, also known as p40 ribosomal protein and RPSA, acts as an integral component of the 40S ribosomal subunit [19] and is involved in cellular translation and proliferation [20]. LamR is highly conserved across species from bacteria to humans [21]. The LamR orthologs in yeast have been shown to be polysome-associated [22] and involved in maturation of the 40S ribosome, specifically processing of the 20S to 18S rRNA [23]. In addition, LamR plays a role in maintaining cell viability in yeast [23] and in a number of human cells [20], [23], [24], [25], [26].

Previous studies have also implicated 67 kDa LamR in binding interactions with actin at the cell membrane. A 70 kDa cell-surface protein, originally called connectin, was found to bind both laminin and actin *in vitro* [27]. It was also found that clustering of laminin in the ECM results in LamR clustering and subsequent actin remodeling [28]. Further, a 69 kDa laminin-binding protein was found to interact with microfilaments to mediate cell attachment and migration [29]. These data indicate that LamR interactions with the cytoskeleton might play a role in cell motility.

The cytoskeleton, an elaborate network of proteins, is responsible for providing structure and shape to the cell and manipulating the cell membrane to induce cell motility. This network is comprised of three main types of proteins: microfilaments comprised of helical assemblies of actin, microtubules comprised of alpha and beta tubulin dimers and intermediate filaments comprised of a number of different proteins, depending on cell type. The cytoskeleton is also associated with many cellular components such as the nucleus, the cell membrane, vesicles and other macromolecules [30], [31], [32], [33]. This protein meshwork acts as a highway connecting different points of the cell and utilizing molecular motors powered by filament assembly forces to transport proteins and organelles across the cell's span [34], [35], [36]. In response to migration-inducing stimuli, actin repolymerizes, polarizing the cell and enabling the formation of lamellipodia and filapodia protrusions [37]. These protrusions, which are stabilized by transmembrane receptors interacting with the ECM, enable the cell to crawl by the use of these adhesions at the leading edge [38]. In addition, the cytoskeleton plays a role in cellular translation. It was originally thought that translation of select transcripts occurred at the cytoskeleton [39], however new evidence indicates that a significant portion of translation may occur bound to the cytoskeleton [40]. Immunofluorescence staining and electron microscopy indicates that polysomes co-localize with cytoskeletal components [41], [42], [43], [44], [45]. Detergent treatment of cells, which removes polysomes bound to the endoplasmic reticulum, indicates that polyribosomes bind to the cytoskeleton [42], [46], [47]. Treatment with agents that depolymerize actin or induce improper organization causes the release of polysomes from the cytoskeleton and inhibits protein synthesis [42], [48], [49], [50].

This study examines LamR interactions with the cytoskeleton. Utilizing microscopy we have shown that the interaction between LamR and F-actin is related to LamR functions at the cell membrane. Microscopy revealed actin reorganization, formation of lamellipodia, and LamR localization at the leading edge following cell plating on laminin. Ablation of LamR expression inhibited cell migration comparable to the effects of inhibiting actin filament formation. Further, 37 kDa LamR was found to directly bind actin in an *in vitro* binding assay. These data indicate that the interaction between actin and LamR mediates cell motility.

Through microscopy we have found that LamR and S6, another 40S ribosomal component co-localize with α-tubulin. Co-localization is lost with treatment that disrupts microtubule dynamics. The direct interaction of LamR with tubulin, coupled with the dissociation of S6 from the cytoskeleton following treatment with taxol or siLamR, suggests that LamR may act as a tether to bind the translation complex to the cytoskeleton. This study demonstrates that interactions between LamR and components of the cytoskeleton, actin and tubulin, play a role in mediating cell motility and translation.

## Results

### LamR Localization

**Figure 1:**
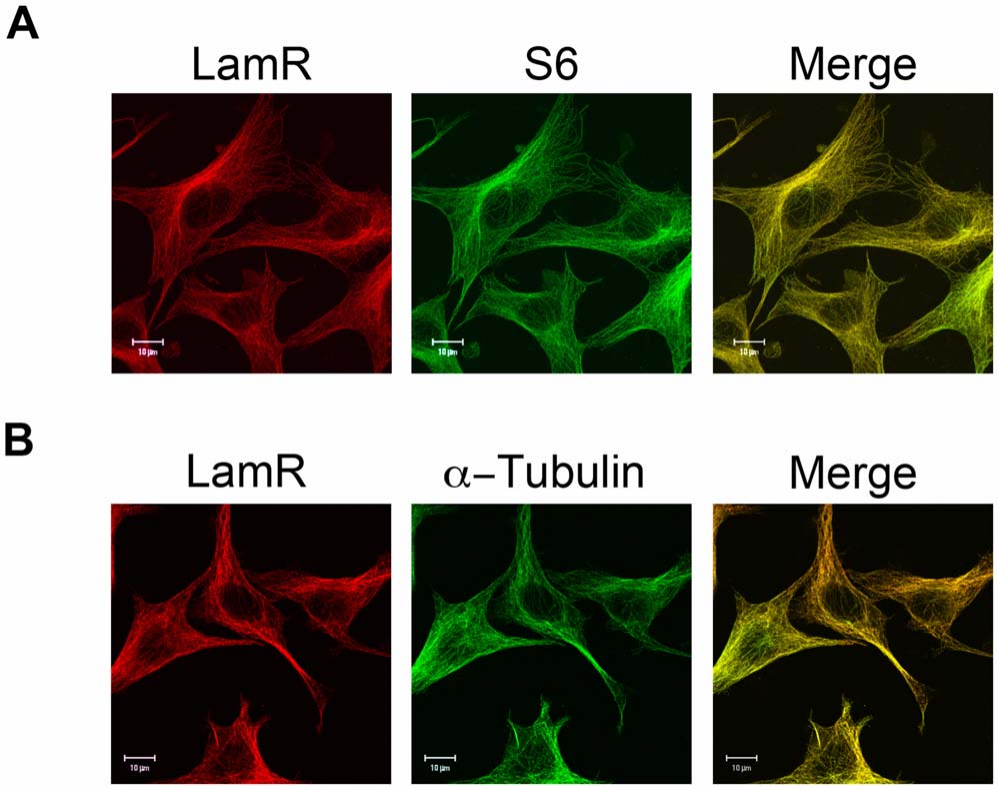
The S6 ribosomal protein and α-tubulin co-localize with LamR. (A) Immunofluorescence of fixed NIH 3T3 cells stained for LamR (H141) (left), S6 (middle) and a merged image (right) illustrate co-localization of S6 with LamR. (B) Immunofluorescence of a fixed specimen stained for LamR (H141) (left), α-tubulin (middle) and the merged image (right) indicate the co-localization of LamR with α-tubulin. Scale bars in A and B represent 10 µm.

LamR is an integral ribosomal component, which is required for protein translation [19], [20]. Through the use of immunofluorescence, we confirmed co-localization of LamR with S6, used throughout this study as a marker for the 40S ribosomal subunit (Figure 1A). In order to study LamR interactions with cytoskeletal components, we examined the cellular localization of α-tubulin in relation to LamR. These data revealed that LamR also co-localizes with α-tubulin (Figure 1B), which indicates that LamR in complex with the ribosome may be associated with tubulin.

### Characterization of the LamR-α-Tubulin Interaction

**Figure 2 pone-0015895-g002:**
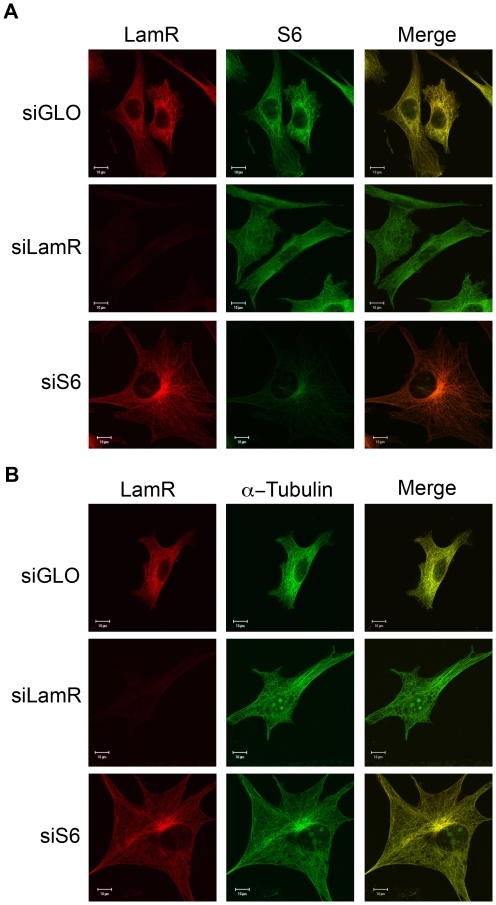
LamR tethers the ribosome to the cytoskeleton. (A and B) Immunofluorescence following knockdown of either LamR or S6. (A) Microscopy image of samples stained for LamR (H141) (left), S6 (middle) or a merged overlay (right). Knockdown of LamR results in a change of the S6 localization from cytoskeleton-associated to diffuse. Knockdown of S6 has minimal effect on LamR localization, indicating that LamR tethers S6 to the cytoskeleton. (B) Immunofluorescence of samples stained for LamR (H141) (left), α-tubulin (middle) or merged images (right). Microscopy indicates that α-tubulin localization remains unaffected by knockdown of either protein. Scale bars in A and B represent 10 µm.

To study interactions between the ribosome and α-tubulin, siRNA was employed to ablate expression of either LamR or S6 ribosomal protein. LamR-specific siRNA successfully ablates expression of both the 37 and 67 kDa forms of the protein. Successful knockdown of LamR and S6 was confirmed by western blot analysis (Figure S1A and B). Although knockdown of S6 expression was less efficient than LamR, ^35^S labeling confirmed inhibition of protein synthesis (Figure S1C). siGLO, a RISC free fluorescently conjugated control oligo, coupled with FACS analysis was utilized to assess transfection efficiency (Figure S1D). siLamR and siS6 treated samples were subjected to immunofluorescence to study the effect knockdown had on association with α-tubulin. In cells where expression of LamR had been ablated, cytoplasmic S6 staining became diffuse, indicating that S6 was no longer associated with α-tubulin (Figure 2A, middle panels). Additionally, siLamR induced accumulation of S6 in the nucleus. LamR plays a role in ribosome maturation [23], which could be inhibited in siLamR treated cells. Since some ribosomal proteins have been shown to bind to pre-ribosomes in the nucleus [51], loss of ribosome maturation may cause these ribosomal proteins to accumulate in the nucleus. This may account for the accumulation of S6 in the nucleus in siLamR treated cells. In cells where expression of S6 had been ablated, no change in LamR's cytoplasmic localization was observed, indicating that S6 is not required for LamR association with α-tubulin (Figure 2A, bottom panels). Treatment with siS6 did induce the accumulation of LamR in the perinuclear region possibly near the microtubule organizing center, which could be the result of S6-induced cell cycle arrest. Knockdown of either LamR or S6 had no effect on cytoplasmic α-tubulin localization (Figure 2B), however siLamR treatment did result in the accumulation of α-tubulin in the nucleus. Tubulin shuttles between the nucleus and cytoplasm [52] and the cell cycle arrest induced by siRNA treatment could induce its accumulation in the nucleus. These data indicate that LamR is required for components of the 40S ribosomal subunit to co-localize with α-tubulin.

### Translation and the Cytoskeleton

**Figure 3:**
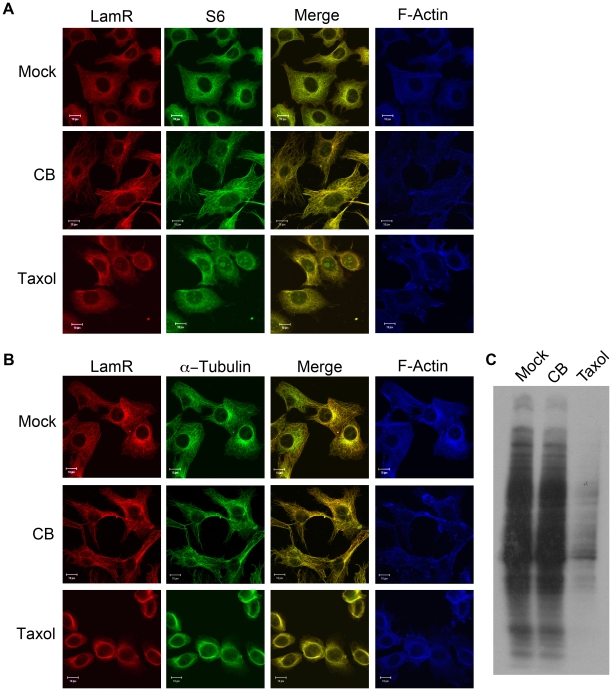
The cytoskeleton is important for translation. (A) Treatment of cells with agents to disrupt normal dynamics of the cytoskeleton induces changes in LamR localization. NIH 3T3 cells treated with either CB to disrupt the actin filaments or, taxol to block tubulin dynamics were subjected to immunofluorescence. Samples were stained for LamR (H141) (left) or S6 (middle) with the merged image. While treatment with CB had no effect, taxol treatment produced a diffuse staining pattern for both LamR and S6, indicating that these proteins dissociated from the cytoskeleton. (B) Samples treated similarly to panel A, were stained with LamR (H141) (left) or α-tubulin (middle) and overlaid image. In A and B panels showing F-actin staining (right) were included as confirmation that the CB treatment was successful. Scale bars in A and B represent 10 µm. (C) ^35^S labeling of cells following treatment with either CB or taxol. Protein synthesis was inhibited in taxol treated samples, however CB treatment had no effect.

Our data demonstrating the co-localization of components of the 40S ribosome with α-tubulin indicates that some cellular translation occurs at the cytoskeleton (Figure 1). To assess the requirement of different components of the cytoskeleton for cellular translation cells were treated with either cytochalasin B (CB), which blocks monomer addition to actin filaments, or taxol, which stabilizes microtubules and inhibits tubulin dynamics. Treatment with CB resulted in loss of actin filament structure, but had no effect on LamR or S6 localization (Figure 3A, middle panels). While CB treatment had no effect on the α-tubulin localization, taxol treatment resulted in an alteration of tubulin structure (Figure 3B). The alteration in tubulin structure was likely the result of a long incubation with taxol and not a loss of cell viability, which was monitored in parallel. Treatment with taxol also had a dramatic effect on both LamR and S6 localization, causing both proteins to adopt a diffuse staining pattern (Figure 3A, bottom panels).

The association between α-tubulin and 40S ribosomal components S6 and LamR indicates that this interaction is related to cellular translation. To further characterize the relationship between tubulin and protein synthesis, cells were treated with either CB or taxol and subjected to ^35^S labeling. Interestingly, there was no change in protein synthesis in cells treated with CB (Figure 3C). However, in cells treated with taxol, new protein synthesis was significantly decreased (Figure 3C). These data indicate that tubulin plays a critical role in mediating cellular translation.

### Characterization of the LamR-Actin Interaction

**Figure 4:**
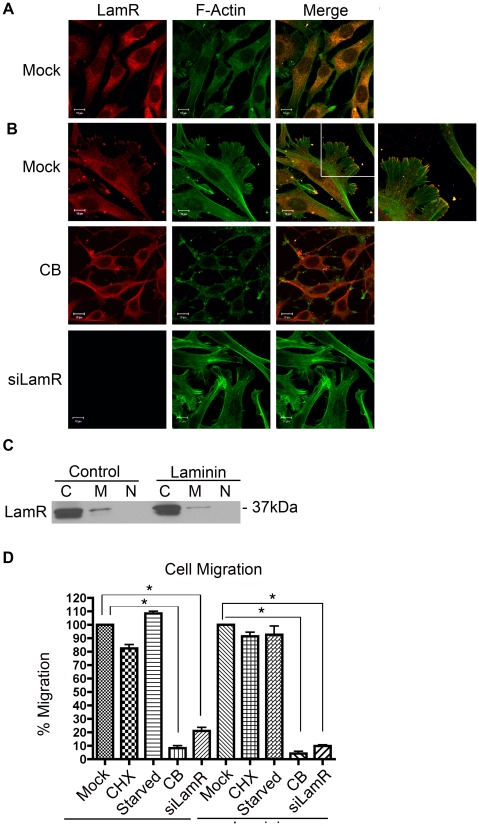
Characterization of the interaction between LamR and F-actin. (A) Immunofluorescence of NIH 3T3 cells stained for LamR (F18) (left) or F-Actin (middle) and merged (right). (B) Plating cells on laminin-coated plates induces lamellipodia formation and LamR localizes to the terminal ends of the actin fibers. Mock treated NIH 3T3 cells (top), cells treated with CB (middle) or cells transfected with siLamR (bottom) were plated on laminin coated chamber slides. Cells were processed for immunofluorescence, staining for LamR (F18) (left) or F-actin (middle) and merged (right). The additional panel shown with the mock-treated sample represents a zoomed image of the area indicated by the white square. Following treatment with CB, lamellipodia formation is inhibited, however, after knockdown of LamR these structures still form but LamR is not available to localize to the actin fibers. Scale bars in A and B represent 10 µm. (C) Culturing cells on laminin-coated plates had no effect on localization or concentration of LamR within the samples. Fractionation of cells plated on tissue culture coated or laminin coated plates. Samples were separated into cytosolic (c), membrane (m) and nuclear (n) fractions. (D) Treatment with CB or ablation of LamR expression reduces cell migration. A graphical representation of the ability of NIH 3T3 cells treated with CB or transfected with siLamR to migrate toward 10% FCS or laminin. CHX and serum starved samples serve as controls for translation inhibition and cell cycle arrest respectively. Data in D represents the SEM (error bars) of three experiments. Each sample was corrected for percent viability and was compared to the mock treated control. Statistical significance was calculated by a two-tailed student t-test (* P<0.005).

LamR interactions with tubulin appear to mediate translation and are concentrated within the intracellular environment (Figures 1–3). Interactions between LamR and actin appear to affect extracellular functions. Under normal cell culture conditions, LamR did not co-localize with F-actin at the cell surface (Figure 4A). Plating cells on laminin coated chamber slides induced a change in both cell morphology and LamR localization (Figure 4B, top panels). The presence of laminin on the culture dish induced the reorganization of actin filaments and the formation of lamellipodia with LamR concentrated at the terminal ends (Figure 4B, top panels). Cellular fractionation coupled with western blotting illustrated that there was no change in LamR localization or concentration when cells were cultured on laminin (Figure 4C), suggesting that the altered immunofluorescence pattern resulted from a redistribution of LamR within the cellular compartments. The additional band in the cytoplasmic fraction likely represents LamR prior to post-translational modification, which is important for membrane localization.

To study the formation of lamellipodia structures, cells were treated with CB or LamR-specific siRNA to prevent formation of actin filaments and translation of LamR, respectively. Treatment with CB resulted in a loss of lamellipodia (Figure 4B, middle panels). Ablation of LamR expression did not inhibit lamellipodia formation (Figure 4B, bottom panels). Treatment with siLamR or CB indicates that lamellipodia formation is dependent on a functional actin structure rather than LamR.

The formation of lamellipodia is indicative of cell migration [53]. To determine if both of these proteins are required for cell migration, cells were either treated with CB or transfected with siLamR and their ability to migrate to either purified laminin or 10% fetal calf serum (FCS) was assessed. Following either treatment migration was reduced by 80% (Figure 4D). To confirm that LamR expression and not inhibition of translation or cell cycle arrest was responsible for the migration inhibition, cells were treated with cycloheximide (CHX) or serum starved, respectively. The efficacy of CHX treatment or serum starvation was confirmed by ^35^S labeling and propidium iodide staining respectively (Figure S2). Both CHX treatment and G1 phase cell cycle arrest had no effect on cell migration indicating that the reduction in migration was specific to the loss of LamR expression. These data indicate that both LamR and actin play an important role in cell migration.

### LamR Binds to Components of the Cytoskeleton

**Figure 5:**
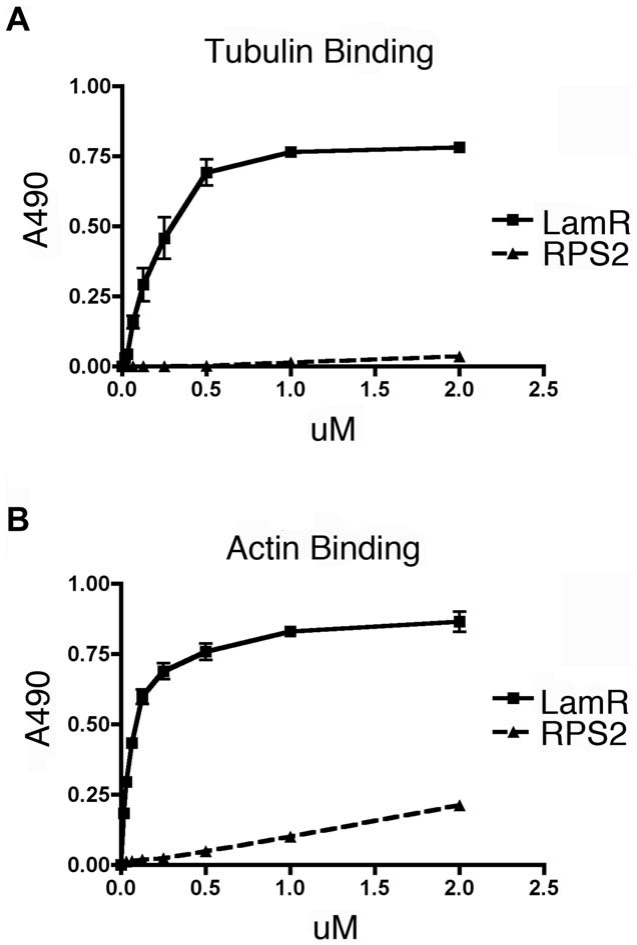
LamR binds directly to components of the cytoskeleton. (A and B) LamR binds directly to the cytoskeleton as shown by an *in vitro* ELISA assay whereby purified microtubules (A) or filamentous actin (B) have been coated onto plates and the ability of purified recombinant LamR to bind was assessed. Graphical representations of the binding of either LamR (solid line) or ortholog, *A. fulgidus* S2p (RPS2) (dashed line). LamR is able to bind directly to both tubulin and actin at a significantly higher affinity than RPS2. Data in A and B represents the SEM (error bars) of three experiments.

Immunofluorescence data indicate that LamR interacts with tubulin (Figures 1–3) and both immunofluorescence and migration data (Figure 4) demonstrate that LamR interacts with actin. To determine whether LamR- cytoskeleton interactions are direct binding events, we utilized purified recombinant LamR in a binding enzyme-linked immunosorbent assay (ELISA) to measure binding activity *in vitro*. Either tubulin or actin was immobilized on an ELISA plate and LamR binding activity was assessed (Figure 5A and B). A bacterial ortholog of LamR, *A. fulgidus* S2p ribosomal protein (RPS2), was used as a negative control. LamR exhibits specific binding to both tubulin and actin compared with *A. fulgidus* S2p. LamR binding affinity was in the low micromolar range, which suggests that interactions between LamR and both tubulin and actin are of high affinity.

This study demonstrates that interactions between LamR and tubulin or actin mediate both intracellular and extracellular functions. Together these data indicate that LamR directly interacts with components of the cytoskeleton and that these interactions are important for mediating cellular translation and migration.

## Discussion

LamR plays a role in intracellular functions, such as translation, through its role as a component of the 40S ribosome [19], [20] and extracellular functions, such as cell migration and adhesion, through its role as a cell surface receptor [2], [17]. Previously, interactions between LamR and the cytoskeleton, specifically actin, were thought to be exclusively related to cell motility and attachment [29]. In addition, cytoskeletal interactions with the ribosome have been shown, although the role of LamR has not been previously elucidated. The studies presented here demonstrate the direct, high affinity interaction between LamR and both actin and tubulin. These data also suggest that LamR interactions with tubulin are vital to LamR ribosomal functions, specifically protein translation.

Co-localization of ribosomes with cytoskeletal components has been verified by electron microscopy [54] and cell fractionation studies [42]. Previous studies have implicated actin [41], intermediate filaments [43] and tubulin [45], [55] as the cytoskeletal components responsible for ribosomal interactions. A large-scale proteomics study aimed at finding microtubule binding proteins in *Arabidopsis* identified LamR (ribosomal protein S2) [55]. Our study indicates an interaction between LamR and tubulin in mammalian cells, and also provides functional insight into this interaction. Our microscopy studies, showed that LamR and α-tubulin co-localize. Ablation of LamR and S6 expression demonstrated that LamR, but not S6 was critical for interactions between α-tubulin and the ribosome. *In vitro* binding assays verified that LamR specifically binds tubulin with micromolar affinity. Together, these data strongly implicate LamR as the protein responsible for tethering the ribosome to the cytoskeleton.

Translation at the cytoskeleton was originally believed to be responsible for the targeted protein synthesis of a small number of mRNAs [39]. Evidence is now supporting the concept that a large proportion of cellular translation takes place at the cytoskeleton [40]. Through the use of CB and taxol, under conditions where greater than 80% of cells are viable, we were able to inhibit the dynamics of either actin filaments or microtubules, respectively. These manipulations enabled the study of these two cytoskeletal components to identify their specific interactions with LamR. Taxol treatment inhibits tubulin dynamics through the hyperstabilization of microtubules. However, in our studies taxol disrupted the microtubule structure, similarly to treatment with nocodazole. The alteration in tubulin staining following taxol treatment could have resulted from the extended incubation time and was not the result of a loss in cell viability. In addition to the disruption of the tubulin structure, taxol treatment dramatically altered the localization of both S6 and LamR indicating that they are bound to microtubules. Conversely, treatment of cells with CB had no effect on LamR or S6 localization, indicating that neither protein was associated with actin. These treatments were also used in conjunction with ^35^S labeling to study the role of microfilaments and microtubules in translation. These studies revealed that treatment with taxol dramatically inhibits new protein synthesis. Previous studies have indicated that taxol induces translational arrest [56], [57]. These studies confirm taxol-induced translational arrest and indicate that not only is ribosomal association with tubulin vital to translation, but also that a significant amount of translation occurs at the cytoskeleton.

LamR plays an important role in attachment to the ECM. As a cell surface receptor, LamR also plays a role in tumor invasion and metastasis. Previous studies have shown co-localization of F-actin with LamR within the cytoplasm of bovine vascular smooth muscle cells [29]. Our *in vitro* binding studies showed a direct and high affinity interaction between LamR and actin. Additionally, our microscopy studies showed the reorganization of actin filaments when cells are plated on laminin. We have also observed the formation of lamellipodia in which LamR localized at the termini of the actin fibers. Treatment with CB or transfection with siLamR reveals that lamellipodia formation is induced by actin filament reorganization, rather than LamR, since lamellipodia still formed in siLamR transfected samples. Lamellipodia formation is an important step in cell migration during which actin filament reorganization is coupled with interactions with the ECM [53]. Our microscopy data examined the role of both LamR and actin for efficient cell migration. We showed that CB treatment and siLamR transfection independently reduced cell migration to either 10% FCS or purified laminin by 80%, indicating that both proteins are important for cell migration. Through other studies in our lab aimed at characterizing the functions of LamR and its interactions with laminin, it was determined that incubation of cells with purified recombinant LamR can block their ability to migrate toward laminin. Additionally, mutational analysis revealed that the binding sites for actin and tubulin are separate from the binding site of laminin [58]. These data underscore the importance of LamR in cell migration.

**Figure 6:**
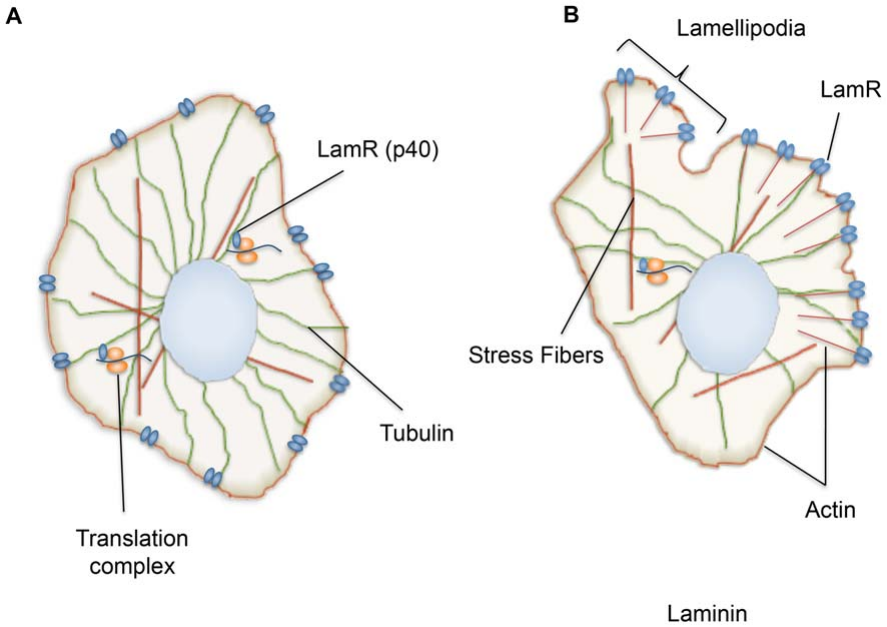
Model depicting the interactions of LamR with the cytoskeleton. (A) LamR's interaction with tubulin is associated with cellular translation. LamR tethers the ribosomal components to tubulin and translation can occur. When cells are plated on poly D-lysine, actin is localized to the membrane and does not co-localize with LamR. (B) When cells are cultured on laminin-coated plates, actin reorganization is induced, resulting in the formation of lamellipodia with LamR concentrated at the terminal ends. The LamR- actin interaction is most likely important for mediating the cell surface functions of LamR, whereas the interaction with tubulin is integral to cellular translation.

We propose a model of interactions between LamR and the cytoskeletal components actin and tubulin (Figure 6). LamR directly interacts with both actin and tubulin, however, each interaction appears to serve a different purpose. LamR interactions with tubulin are critical for cellular translation, where LamR acts as a molecular tether linking the ribosomal components to tubulin (Figure 6A). LamR interactions with actin are more complex. When cells are plated on tissue culture coated or poly D-lysine coated plates, LamR does not co-localize with F-actin at the membrane (Figure 6A). However, when cells are cultured on laminin-coated plates, lamellipodia form and LamR is reorganized to the terminal ends of the actin fibers (Figure 6B). The interaction between LamR and actin is most likely important for the functions of LamR as a cell surface receptor, specifically cell attachment and motility.

LamR is involved in numerous pathologies due to its role in binding virus [8], [9], [10], [59], prion proteins [11] and its overexpression in cancer [12], [13], [14], [15], [16]. The expression of several other ribosomal proteins is elevated in human cancers [60], [61], [62] supporting the link between overexpression of LamR and cellular transformation. LamR is involved in many processes that mediate tumor aggressiveness including, cell migration [17], invasion [63] and ECM remodeling [18]. The correlation between LamR expression and tumor aggressiveness underscores the importance of understanding LamR's diverse functions. This study demonstrates LamR's ability to directly bind tubulin and actin. Through its interaction with tubulin, LamR acts as a molecular tether linking the ribosomal components to tubulin and mediating cellular translation. The LamR-actin interaction is critical for cell migration, indicating that this complex may be important to metastasis. These data indicate that interactions between LamR and the cytoskeleton are vital in mediating two processes that are intimately linked to cancer. Study of the mechanisms of LamR and cytoskeletal interaction may lead to the development of novel anti-cancer therapeutics.

## Materials and Methods

### Cell Lines and Treatments

NIH 3T3 cells were obtained from the American Type Culture Collection. Cells were maintained in Dulbecco's modified Eagle's medium (DMEM) with 10% FCS supplemented with 100 µg/ml of penicillin-streptomycin and 0.5 µg/ml amphotericin B (all from Mediatech).

NIH 3T3 cells were treated with CB (Sigma) at a final concentration of 5 µg/ml for 30 minutes at 37°C to disrupt the actin filament structure. Cells were washed with phosphate buffered saline (PBS) and used for downstream experiments. Cell viability was assessed in parallel to each experiment (as described below) following similar treatment and only samples greater than 80% viable were used.

NIH 3T3 cells were treated with Paclitaxel (taxol) (NovaPlus) at a final concentration of 5 µM for 5 hours at 37°C to inhibit microtubule dynamics. Cells were washed with PBS and used for downstream experiments. Cell viability was assessed in parallel to each experiment (as described below) following similar treatment and only samples greater than 80% viable were used.

NIH 3T3 cells were treated with CHX (Sigma) at a final concentration of 50 µg/ml for 5 hours at 37°C to inhibit cellular translation. For downstream experiments cells were maintained in CHX throughout. Cell viability was assessed in parallel (as described below) following similar treatment and only samples greater than 80% viable were used. For each experiment inhibition of translation was confirmed using ^35^S labeling.

G1 phase cell cycle arrest was induced by incubating NIH 3T3 cells in serum free media for 24 hours at 37°C. Cell cycle arrest was assessed through propidium iodide staining followed by fluorescence activated cell sorting (FACS) analysis. Cell viability was also assessed in parallel (as described below).

### Immunofluorescence

NIH 3T3 cells were cultured on poly-D Lysine coated chamber slides. Poly-D Lysine/laminin coated chamber slides were also used as indicated (BD Bioscience). After CB or taxol treatment described above or transfection with siRNA (as described below) samples were processed for immunofluorescence. Briefly, cells were washed with PBS, fixed with 4% paraformaldehyde and permeabilized with 1% Triton X100. Cells were blocked at room temperature with blocking buffer (0.1% Triton X100 containing 3% bovine serum albumin (BSA)). Slides were incubated with anti-LamR H141 (1∶200) (Santa Cruz), which recognizes amino acids 110-250 of human LamR, to monitor intracellular functions, used for co-staining in all α-tubulin experiments, anti-LamR F18 (1∶200) (Santa Cruz), which recognizes amino acids 245-295 of human LamR, to study cell surface functions, used for co-staining in all F-actin experiments, anti-α-Tubulin (1∶1000) (Cell Signaling Technologies) or anti-S6 (1∶50) (Cell Signaling Technologies) antibodies, all diluted in blocking buffer, overnight at 4°C. Slides were washed with PBS and incubated with Alexafluor 488 conjugated goat anti-mouse (1∶500) (Molecular Probes), Alexafluor 594 conjugated donkey anti-rabbit (1∶500) (Molecular Probes) secondary antibodies and Alexafluor 647 conjugated phalloidin (1∶40), used for F-actin staining, (Molecular Probes). Coverslips were mounted with Prolong Gold Antifade Reagent (Invitrogen). For each experiment, samples stained with secondary antibody alone were processed in parallel to control for non-specific staining. Additionally, for all co-localization studies, samples stained for localization of each protein individually were processed in parallel and the fluorescence of adjacent channels was monitored for bleed through. Samples were visualized with a confocal microscope (Axiovert 100m; Carl Zeiss MicroImaging, Inc.) fitted with a plan-Apochroma 100/1.40 oil DIC objective lens. Images were captured with a DKC-5000 digital camera (Sony) using the LSM510 version 3.2 SP2 software (Carl Zeiss MicroImaging, Inc.). Images were divided into individual channels for single color visualization using Adobe Photoshop CS4.

### Western Blotting

Cell lysates were collected using Mammalian Protein Extraction Reagent (Pierce) supplemented with protease inhibitors (Roche) according to the manufacturer's instructions. Protein content was measured using BioRad D_c_ Protein Reagent according to the manufacturer's instructions (BioRad). Lysates containing 25 µg total protein were run on polyacrylamide gels (BioRad) under reducing conditions. Protein was transferred to polyvinylidine fluoride membrane (Millipore). Membranes were blocked with non-fat dry milk and probed with anti-LamR H141 (1∶2000) (Santa Cruz), anti-S6 (1∶1000) (Cell Signaling Technologies) or β-actin (1∶10,000) (Sigma) antibodies diluted in tris buffered saline, 0.05%Tween, 5% BSA. Proteins were detected using horseradish peroxidase (HRP) conjugated secondary antibodies (Santa Cruz) and then exposed by chemiluminescence (Pierce).

### Short Interfering RNA Studies

To ablate expression of LamR or S6, cells were transfected with siGENOME SMARTpool siRNA (Dharmacon). As a control, as well as a measure of transfection efficiency, siGLO, a fluorescently labeled RISC-free siRNA was used (Dharmacon). Briefly, transfections were performed in 12 well plates with cells at 70% confluency. Each oligo was used at a final concentration of 100 nmol/l. Oligos were incubated with Dharmafect reagent IV (Dharmacon) for 15 minutes at room temperature. Then, 0.8 ml of media was added and the mixture was added to the cells. After 24 hours cells were plated according to downstream experiments. siGLO was used to calculate transfection efficiency through FACS analysis. Only samples with greater than 80% transfection efficiency were used. Efficient knockdown of LamR or S6 was assessed through western blotting.

### FACS Analysis

To assess transfection efficiency with siGLO, FACS analysis was employed. Briefly, cells were trypsinized at 37°C and centrifuged at 300× g for 5 minutes at 4°C. Samples were washed with PBS and then resuspended in PBS: 4% paraformaldehyde (1∶1) for fixation. Samples were run on a FACSCaliber instrument (Becton Dickinson). Data analysis was performed with FlowJo version 8.2 software (Tree Star, Inc.).

For cell cycle analysis following serum starvation cells were trypsinized at 37°C and centrifuged at 300× g for 5 minutes at 4°C. Samples were washed with PBS and resuspended in PBS. Ice cold 100% ethanol was added dropwise and RNase was added at a final concentration of 100 µg/ml. Samples were incubated overnight at 4°C. Samples were then centrifuged at 300× g for 5 minutes at 4°C and washed with PBS. Samples were resuspended in PBS with the addition of propidium iodide at a final concentration of 50 µg/ml. Samples were run on a FACScan instrument (Becton Dickinson). Data analysis was performed with ModFit LT v3.0 software (Verity Software House, Inc.).

### ^35^S Labeling

Following CB or taxol treatment, as described above, cells were incubated with ^35^S methionine/cysteine (20 µCi/ml) (Perkin Elmer) in methionine-free media for 2 hours at 37°C. Cells were washed to remove unbound label and then incubated in DMEM supplemented with 10% FCS for 30 minutes. Lysates were collected with Mammalian Protein Extraction Reagent (Pierce) and samples containing 20 µg of total protein were run on a 4–15% gradient gel (BioRad). The gel was fixed in a solution of 50% methanol and 10% acetic acid for 30 minutes with agitation. The gel was then incubated with enhancer solution (GE Healthcare) for 10 minutes, dried for 2 hours at 80°C under vacuum and exposed to film overnight at −80°C.

### Assessment of Cell Viability

Cells were cultured on 96 well luminescence plates (BD Bioscience). After initial treatment with CB, taxol, CHX, serum starvation or siRNA transfection, cell viability was assayed with the Cell Titer Glo assay (Promega). Briefly, Cell Titer Glo reagent was added 1∶1 directly to the cell culture media. Samples were incubated at room temperature for 2 minutes with agitation and then for an additional 10 minutes. After incubation luminescence was read with a multiwell plate reader, Wallac EnVision (Perkin Elmer). To calculate cell viability each sample was compared with a similarly treated mock control.

### Cell Fractionation

NIH 3T3 cells were cultured on tissue culture plates or laminin coated plates (BD Bioscience). Cells were trypsinized, washed with PBS and resuspended at 5×10^6^ cells/ml. Cells were subjected to fractionation to separate cytosolic, membrane and nuclear fractions using the Qproteome Cell Compartment Kit (Qiagen) according to the manufacturer's instructions. Successful fractionation was confirmed by western blotting with markers specific to each fraction.

### Cell Migration Assay

Cells transfected with siRNA or untreated NIH 3T3 cells were used for these assays. Transfected cells were harvested in the optimal knockdown period, as confirmed by western blotting done in parallel. Cell viability was also tested in parallel (as described above). siRNA transfected, CHX, serum starved, CB or mock treated cells were tested for their ability to migrate to either 10% FCS or 15 µg/ml purified laminin (Invitrogen) using the CytoSelect™ 24-Well Cell Migration Assay (8 µm, Colorimetric Format) (Cell Biolabs, Inc.). Briefly, 1.5×10^5^ cells in unsupplemented media were added to the upper insert chamber of each well with the reservoir below containing 500 µl of either 10% FCS, purified laminin or unsupplemented media (used to calculate assay background). The plate was incubated at 37°C for 5 hours. After the incubation was completed the upper membrane of each insert was thoroughly washed with dH_2_O to remove non-migratory cells. Inserts were then incubated in cell stain solution for 10 minutes at room temperature, washed again in dH_2_O, and allowed to dry. Inserts were then incubated with extraction solution at room temperature for 10 minutes with gentle agitation. 100 µl of each sample was transferred to an ELISA plate and read at 630 nm with the ELx800 plate reader (Biotek Instruments, Inc.). To calculate migration ability, background was subtracted and then each sample was corrected for cell viability and compared to the similarly treated mock sample.

### Protein Purification

Recombinant LamR was purified as described previously [64]. Briefly, a construct generated from human full length LamR was transformed into E.coli strain BL21 (DE3*) and grown in Luria broth to OD_600_ of 0.6 at 37°C. Protein expression was induced by the addition of isopropyl-thiogalactopyranoside, 0.1 mM, at 20°C. Cells were harvested and lysed by French press. The lysate was cleared by centrifugation at 16,000 RPM for 30 minutes. The supernatant was purified in two steps: by Ni-NTA chromatography (Qiagen) followed by gel filtration chromatography (Superdex 75) (Amersham). *A. fulgidus* S2p (RPS2) (residues 1–208) was expressed and purified under the same conditions.

### Protein Binding ELISA

ELISA plates (Costar) were coated with 1.5 µg per well of either filamentous actin or preformed microtubules (Cytoskeleton Inc.) dissolved in coating buffer (0.1 M sodium bicarbonate pH 9.2) overnight at 4°C. Plates were washed with wash buffer (PBS containing 0.5% Tween). Plates were then incubated in blocking buffer (2% FCS, 1 mg/ml BSA, 0.1% sodium azide in PBS) for 1 hour at 37°C. After incubation, plates were washed with wash buffer, 3 times for 5 minutes each. Triplicate wells were incubated with indicated concentrations of either purified LamR or *A. fulgidus* S2p (RPS2) for 1 hour at 37°C. Plates were washed with wash buffer. Wells were incubated with Penta-His HRP conjugate (Qiagen) for 2 hours at room temperature. Plates were washed and substrate solution was added. Absorbance at 490 nm was measured on the ELx800 ELISA plate reader (Biotek Instruments, Inc.) Each sample was normalized to background absorbance and a binding curve was generated with Prism4.

## Supporting Information

Figure S1 **siRNA treatment controls.** (A and B) Western blot analysis of lysates collected from siLamR (A) or siS6 (B) transfected NIH 3T3 cells illustrates efficient ablation of protein expression. In A and B βactin was used as a loading control. (C) ^35^S labeling of siRNA transfected samples. Protein synthesis remains unaffected in the siGLO-transfected, control sample. (D) Transfection efficiency was monitored through transfection of siGLO, a fluorescently labeled, RISC-free control oligo and quantified by FACS analysis.
(TIF)

Figure S2 **CHX and serum starvation treatment controls.** (A) ^35^S labeling of CHX treated NIH 3T3 cells. CHX inhibited protein synthesis relative to the mock treated control. (B) Cell cycle profile of mock and serum starved samples. Serum starvation efficiently induced G1 phase cell cycle arrest.
(TIF)
