# Supplementary figures and images for: Interactions Between Laminin Receptor and the Cytoskeleton During Translation and Cell Motility
Source: PLoS One. 2011 Jan 7;6(1):e15895. doi: 10.1371/journal.pone.0015895 (PMC3017552; doi:10.1371/journal.pone.0015895)

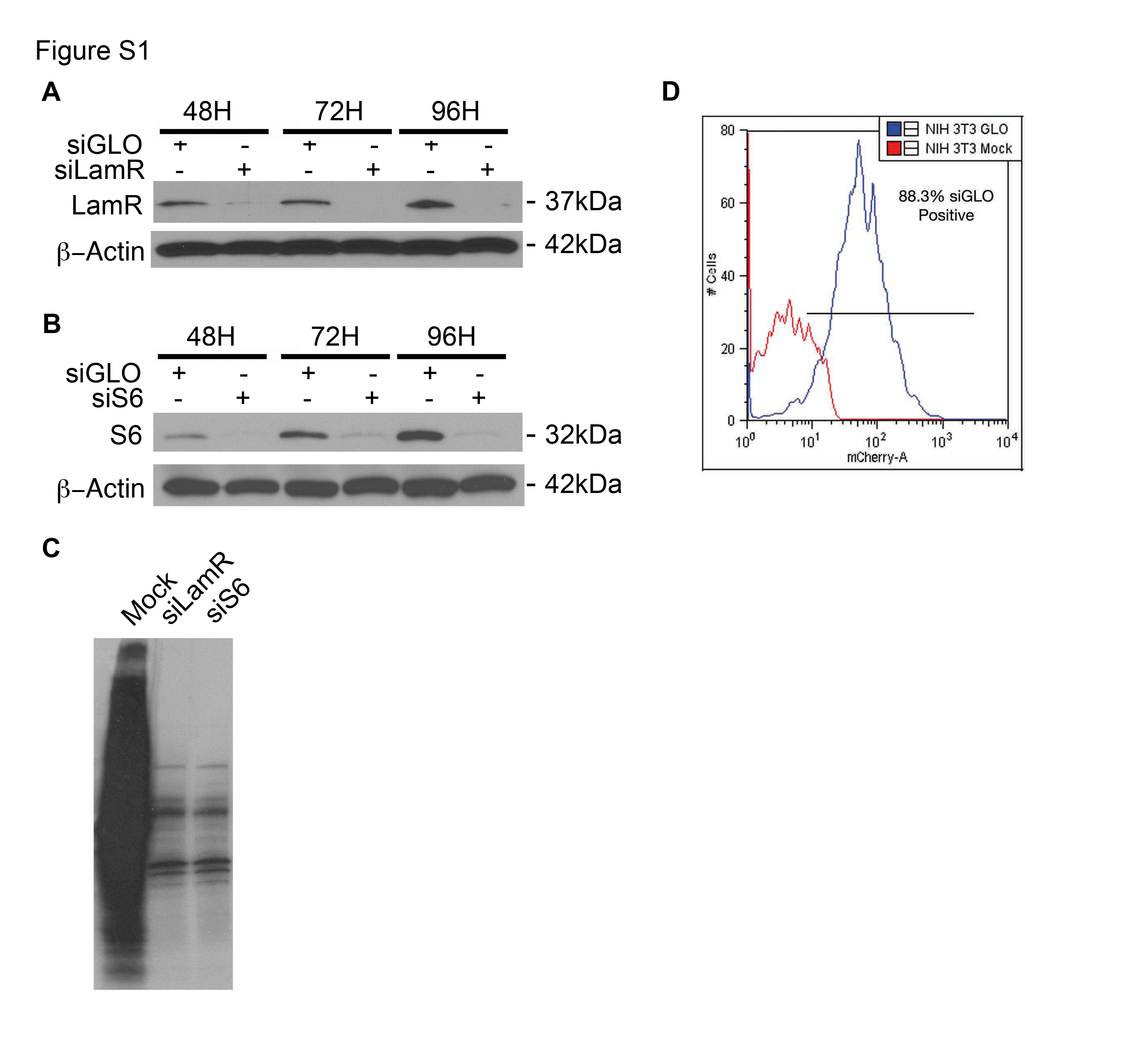

Supplement: Figure S1 — siRNA treatment controls. (A and B) Western blot analysis of lysates collected from siLamR (A) or siS6 (B) transfected NIH 3T3 cells illustrates efficient ablation of protein expression. In A and B βactin was used as a loading control. (C) 35S labeling of siRNA transfected samples. Protein synthesis remains unaffected in the siGLO-transfected, control sample. (D) Transfection efficiency was monitored through transfection of siGLO, a fluorescently labeled, RISC-free control oligo and quantified by FACS analysis. (TIF) [file pone.0015895.s001.tif]

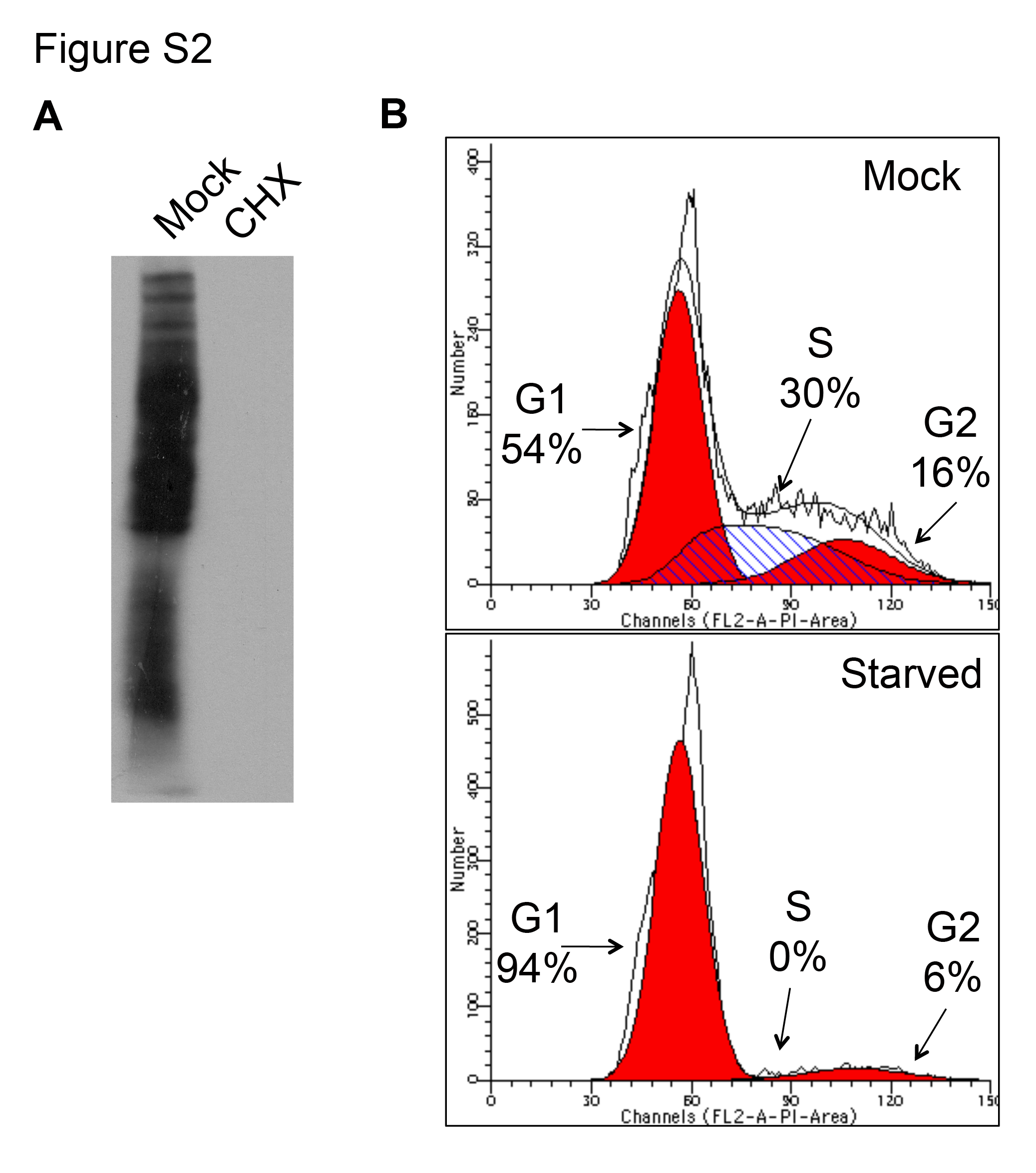

Supplement: Figure S2 — CHX and serum starvation treatment controls. (A) 35S labeling of CHX treated NIH 3T3 cells. CHX inhibited protein synthesis relative to the mock treated control. (B) Cell cycle profile of mock and serum starved samples. Serum starvation efficiently induced G1 phase cell cycle arrest. (TIF) [file pone.0015895.s002.tif]
